# Opening of an Institution for the Treatment of Idiocy in Edinburgh

**Published:** 1855-10-01

**Authors:** 


### OPENING OF AN INSTITUTION FOR THE TREATMENT OF IDIOCY IN EDINBURGH.

Hitherto only one institution has existed in Scotland for the treatment and training of imbecile and idiot children; that, namely, which was founded two years ago by Sir John and Lady Jane Ogilvy, at Baldovan, near Dundee. It is with pleasure we announce that another is nearly ready for opening in Edinburgh.

So much of the hoped-for success must depend upon the careful adaptation of the physical treatment to the circumstances of each case, that it appears essential to the efficient working of such institutions that they be superintended, if not conducted, by well-qualified medical men. We are, therefore, happy to learn that the Edinburgh School will have the aid of Drs. John Smith and Coldstream, as physicians; while the immediate management and superintendence will be in the hands of Dr. David Brodie. It will be, we trust, the care of these gentlemen to establish a psychical sanatarium for the young, worthy of the metropolis of Scotland, and of the Medical School of Edinburgh.

It is, indeed, rather a humiliating consideration that, whereas it was in Edinburgh that the first institution that ever existed for the education of the deaf and dumb, was established (in 1760 by Mr. Thomas Braidwood), we have been so tardy in commencing operations for the benefit of a section of society so long neglected, but so much in need of such aid as science can give, and this notwithstanding the encouraging results reported as having been obtained elsewhere. Our dilatoriness in this matter is all the more remarkable, seeing that it was in Edinburgh that the first earnest appeal on behalf of the neglected idiot was published, and that so long ago as in 1819. In his interesting Essay on Education, which first appeared in that year as an article in the *Encyclopædia Edinensis*, and subsequently (in 1825) as a separate treatise, Dr. Richard Poole thus wrote :—"It is obvious that there is ground for employing medical advice in cases of general imbecility presenting in early life; and there cannot be a doubt that cases of this kind, which are allowed by despair to become confirmed and deteriorated, might have been relieved by professional interference. The correctness of the principles on which Dr. Poole ventured to found so strong an asseveration, has latterly been fully proved by the experience obtained in several institutions established for the treatment of congenital idiocy; the general result being, that, under suitable means, almost all are improveable; and that "for three-fourths of the children treated, much may be done to rouse intelligence, and even to fit for usefulness."

This unlooked-for result has stimulated some of the European governments, and several benevolent individuals in different countries, to exert themselves on behalf of the poor idiot. The existing institutions at Redhill and Highgate, near London, at Colchester and at Bath, are probably known to most of our readers; also the schools for adult idiots at Hanwell, in England, and Bicetre, at Paris (not to speak of the world-renowned hospice of the Abendberg, conducted by Dr. Guggenbühl, whose success in treating cretinism has gone far to excite a general interest in this subject); but it is perhaps not so generally known, that some distinguished members of the medical profession in Denmark, Prussia, Saxony, and Wurtemburg, have, more or less recently, devoted themselves to the forwarding of this good work in various ways. For example, in Copenhagen, the learned Professor of Physiology in the University, Dr. Eschricht, published last year an interesting treatise "On the Possibility of Educating Idiotic Children to become useful Members of Society." In the same city, Dr. Hybertz has published an elaborate statistical inquiry into the extent to which idiocy prevails in the various countries of Europe, and has also devoted himself to the treatment of a certain number of childrer affected with it. Still, in the Danish capital, M. Moldenhawer has commenced a work of the same kind; while, in Schleswig, Dr. Hansen is similarly employed. Dr. Sägert of Berlin, Director of the Royal Institution for the Deaf and Dumb in that city, has written much and well on the treatment of idiocy, and has for several years laboured personally in training imbecile children. At Bendorf, near Coblenz, Dr. Erlenmeyer has a small establishment of the same kind. In Saxony, Dr. Kern at Leipsic, and Dr. Gläsche at Hubertsburg, near Dresden; in Wurtemberg, Dr. Müller at Winterbach, and Dr. Zimmer at Mariaberg—are all in charge of institutions for treating idiocy, varying in extent.* In the same way the work is extending in the United States of America. There are now, we believe, at least four establishments (one in each of the States of Massachusetts, New York, Pennsylvania, and Virginia) supported at the public expense, besides private ones. The reports of that at Boston, published by Dr. Howe, are of great interest and value.

A proof of the zeal in this good cause which exists in Denmark has lately been shown in the mission of a gentleman, in all respects well qualified, charged by the Government of that country to visit, and report upon, all the institutions for the cure of idiocy in Europe. This commissioner (Mr. John Moldenhawer, who published last year an account of the German establishments), after having visited the English schools, came to Edinburgh in expectation of seeing something worthy of his attention, and was disappointed. Dundee alone, in all Scotland, could furnish him with the material for his report.

The subject of the treatment of idiocy lately engaged the attention of the Academy of Medicine at Paris; a paper on it having been read before that body, in July last, by M. Delasiauve, physician in charge of the epileptics and idiots at Bicètre. This author homologates the axiom of Voisin, regarding the object of the education of the idiot,—namely *to develop what already exists.* He announces a classification of idiots with reference to their various degrees of aptitude for education, and suggests some improvements, both of a general and of a special kind, which he thinks ought to be introduced into establishments devoted to the cure of idiocy.

The above narration will serve to prove how earnestly our brethren in other lands are endeavouring to carry into practical operation the idea of its being possible to rescue the poor idiot from that hopeless degradation and neglect to which he has hitherto been consigned. Let us now, although late, do what we can to emulate the good example thus set us.

To return to our projected Edinburgh Institution, we have further to state, that, as it is desirable to make it self-supporting, the lowest rate of board to be charged will be forty-five pounds *per annum*, and for the first-class accommodation, the charge will be seventy-five pounds. It is expected that the house (which is No. 10, Gayfield Square) will be ready for the reception of pupil-patients in the beginning of October.

The prospectus bears that,

"The institution will receive a certain proportion of children and youth not affected with mental imperfection or peculiarity, but who are, from bodily ailments or other causes, unable to take their place at ordinary schools. The combination which this Institution presents, of practical medical experience with efficient educational resources, will supply, it is hoped, a want which is much felt by the parents of children in the condition here referred to."

We cordially recommend this infant institution to the favourable regards and support of our readers.—*Edinburgh Medical Journal* for September.

* If to this list be added the establishments of Pastor Frobst, at Exberg, in Bavaria, and of Pastor Bost, at Laforce, (Dordogne,) in France, we have a tolerably complete enumeration of the institutions for the benefit of idiotic children, now existing in Europe.

